# Risk Factors Contributing to Higher Mortality Rates in Elderly Patients with Acute Traumatic Subdural Hematoma Sustained in a Fall: A Cross-Sectional Analysis Using Registered Trauma Data

**DOI:** 10.3390/ijerph15112426

**Published:** 2018-11-01

**Authors:** Ching-Hua Hsieh, Cheng-Shyuan Rau, Shao-Chun Wu, Hang-Tsung Liu, Chun-Ying Huang, Shiun-Yuan Hsu, Hsiao-Yun Hsieh

**Affiliations:** 1 Department of Plastic Surgery, Kaohsiung Chang Gung Memorial Hospital, Chang Gung University and College of Medicine, Kaohsiung 83301, Taiwan; sylvia19870714@hotmail.com; 2 Department of Neurosurgery, Kaohsiung Chang Gung Memorial Hospital, Chang Gung University and College of Medicine, Kaohsiung 83301, Taiwan; ersh2127@cloud.cgmh.org.tw; 3 Department of Anesthesiology, Kaohsiung Chang Gung Memorial Hospital, Chang Gung University and College of Medicine, Kaohsiung 83301, Taiwan; shaochunwu@gmail.com; 4 Department of Trauma Surgery, Kaohsiung Chang Gung Memorial Hospital, Chang Gung University and College of Medicine, Kaohsiung 83301, Taiwan; htl1688@yahoo.com.tw (H.-T.L.); junyinhaung@yahoo.com.tw (C.-Y.H.); ah.lucy@hotmail.com (S.-Y.H.)

**Keywords:** traumatic brain injury, subdural hematoma (SDH), trauma, fall, elderly, young adult, mortality, risk factor

## Abstract

Background: We aimed to explore the risk factors that contribute to the mortality of elderly trauma patients with acute subdural hematoma (SDH) resulting from a fall. Mortality rates of the elderly were compared to those of young adults. Methods: A total of 444 patients with acute traumatic subdural hematoma resulting from a fall, admitted to a level I trauma center from 1 January 2009 to 31 December 2016 were enrolled in this study. Patients were categorized into two groups: elderly patients (*n* = 279) and young adults (*n* = 165). The primary outcome of this study was patient mortality in hospital. The adjusted odds ratio (AOR) with 95% confidence interval (CI) for mortality was calculated according to gender and pre-existing comorbidities. Univariate and multivariate logistic regression analyses were performed to identify factors related to mortality in the elderly. Results: The odds ratio for mortality caused by falls in the elderly patients was four-fold higher than in the young adults, after adjusting for gender and pre-existing comorbidities. In addition, the presence of pre-existing coronary artery disease (OR 3.2, 95% CI 1.09–9.69, *p* = 0.035), end-stage renal disease (OR 4.6, 95% CI 1.48–14.13, *p* = 0.008), hematoma volume (OR 1.2, 95% CI 1.11–1.36, *p* < 0.001), injury severity score (OR 1.3, 95% CI 1.23–1.46, *p* < 0.001), and coagulopathy (OR 4.0, 95% CI 1.47–11.05, *p* = 0.007) were significant independent risk factors for mortality in patients with acute traumatic SDH resulting from a fall. Conclusions: In this study, we identified that pre-existing CAD, ESRD, hematoma volume, ISS, and coagulopathy were significant independent risk factors for mortality in patients with acute traumatic SDH. These results suggest that death following acute SDH is influenced both by the extent of neurological damage and the overall health of the patient at the time of injury.

## 1. Introduction

There has been a worldwide increase in the incidence of fall related injuries, in part caused by the increasing elderly population. In addition, approximately 60% of patients are hospitalized due to injuries sustained in a fall. Falls are the leading cause of hospital admission for traumatic injury in elderly patients [1]. It has been reported that falls are the most common cause of acute subdural hematoma (SDH) [1,2,3]. Of 92,030 patients with SDH in the National Trauma Data Bank in the United States, 55,729 (61%) had sustained injuries from falling [4]. Previous studies have revealed an association between the risk of SDH and falls [4]. In older patients, reduced brain parenchyma has been associated with an increased risk of SDH, which may even occur following minor trauma [5]. We have previously reported that a significantly higher percentage of elderly patients sustained SDH following a fall than comparatively young adult patients (10.1% vs. 8.2%, respectively, *p* = 0.032) [1].

SDH usually results from tears in bridging veins, which cross between the cerebral cortex and the dural sinus [6], or, less frequently, a rupture of the superior cortical arteries [7]. Blood accumulates in the space surrounding the brain parenchyma, between the arachnoid mater and the dura [8]. Increased intracranial pressure caused by a hematoma causes further compression and damage to the delicate brain tissue. As the patients grow older, there is a higher prevalence of comorbidities and increased use of medications, including anticoagulants [9,10] and polypharmacy [11]. This can heighten the risk of bleeding and developing further complications. A reduced physiological reserve and vulnerable central nervous system can further impair the ability of elderly patients to accommodate the stress resulting from an injury [12,13]. In the 1990s, the mortality rate for acute SDH was reported to be as high as 60% [2]. The mortality rate of SDH decreased to a level of 20% around the year 2000 and has fallen as low as 14% within the last decade [2]. However, acute SDH has been reported to be a poor prognostic factor for those patients with a traumatic brain injury [14] and is still associated with a greatly increased probability of death when compared to epidural hematoma (EDH) [15,16]. The IMPACT study found that after controlling for age, Glasgow Coma Score (GCS), motor score and pupil reactions, the presence of SDH doubled the odds of a poor outcome at six months [17]. Worse overall outcomes were particularly common in the elderly [18,19]. There was higher mortality for the elderly following falls (adjusted odds ration [AOR] = 1.9, 95% confidence interval [CI]: 1.06–3.59) compared to young adult patients, after adjusting for pre-existing comorbidities and severity of injury [1]. Therefore, this study was designed to identify the risk factors that contribute to the mortality rate of elderly trauma patients with acute SDH resulting from a fall, in comparison with young adults.

## 2. Materials and Methods

### 2.1. Ethics Statement

This study had been approved by the institutional review board (IRB) of the Kaohsiung Chang Gung Memorial Hospital, a level I regional trauma center in southern Taiwan, with a reference number 201700600B0 [20,21]. The need for informed consent was waived because this is a retrospective study using the Trauma Registry System.

### 2.2. Study Population

This study included all adult patients who sustained an acute traumatic SDH in a fall and were admitted to hospital from 1 January 2009 to 31 December 2016. We included only adult patients aged ≥20 years who had sustained a fall from a standing height (≤1 m). We excluded those patients with incomplete data and those who did not have a computed tomography (CT) image of the brain that was of good enough quality to calculate the hematoma volume in the subdural region (CT information unavailable). Patients transferred from another medical institute without CT images were also excluded. The study population was divided into two groups for the purpose of comparison. The two groups comprised elderly patients aged ≥65 years and young adults aged 20–64 years. The patient information retrieved included: age; sex; comorbidities; injury severity score (ISS), expressed as a median and interquartile range (IQR, Q1–Q3); subdural hematoma volume (mL) from the CT image at admission; surgery (decompression via craniotomy or craniectomy with clot evacuation); reoperation (repeat craniotomy or craniectomy, surgery for brain-related complications such as hydrocephalus or abscess, and restoration of the piece of skull bone); length of stay (LOS) in hospital; admission into an intensive care unit (ICU); and mortality in hospital. Comorbidities included diabetes mellitus (DM); hypertension (HTN); coronary artery disease (CAD), congestive heart failure (CHF), cerebral vascular accident (CVA) and end-stage renal disease (ESRD). Coagulopathy of the patient was defined as an international normalized ratio (INR) > 1.2 in the laboratory test at the emergency room. To calculate intracranial blood volume, the pixel values of the DICOM image files were converted to Hounsfield units (HU), a quantitative scale used to describe radiodensity in CT images. The HU for hematomas ranges from +50 to +80 [22], while air, water and bone have an attenuation value of −1000, 0, and +400 HU, respectively. The pixel values outside the skull bone were excluded from the quantification. The length of the hematoma was defined by measuring the linear distance between the corners of the SDH crescent. The breadth was defined measuring the maximum distance of the hematoma from the inner table of the skull, perpendicular to the length. The depth was determined as the number of slices of visible hematoma multiplied by the slice thickness. The product of the length and breadth was calculated as the area. The top five largest areas in consecutive images were calculated as the mean area of a hematoma. The hematoma volume was calculated as mean area × depth and was expressed in mL. An isolated SDH indicated that there was an SDH but no evidence of other hemorrhages such as EDH, intracerebral hemorrhage (ICH), subarachnoidal hemorrhage (SAH), or intraventricular hemorrhage (IVH) on the brain CT image.

### 2.3. Statistical Analysis

We performed statistical analyses using SPSS 22.0 software (IBM Corp., Armonk, NY, USA). The odds ratios (ORs) of the associated conditions of the patients were presented with 95% CIs. The homogeneity of variance of the continuous variables was first assessed using Levene’s test, followed by one-way analysis of variance (ANOVA) with a Games–Howell post-hoc test, which was used to evaluate the differences between elderly and young adult patients. We expressed the continuous data as mean ± standard deviation. Mortality of patients in hospital was the primary outcome of the study. The adjusted odds ratio (AOR) of mortality was calculated with a 95% CI, taking into accounts gender and pre-existing comorbidities. Univariate and multivariate logistic regression analysis was performed to identify factors related to mortality in the elderly. Results were considered to be statistically significant when a *p* value of <0.05 was obtained.

## 3. Results

### 3.1. Patient and Injury Characteristics

**Figure 1:**
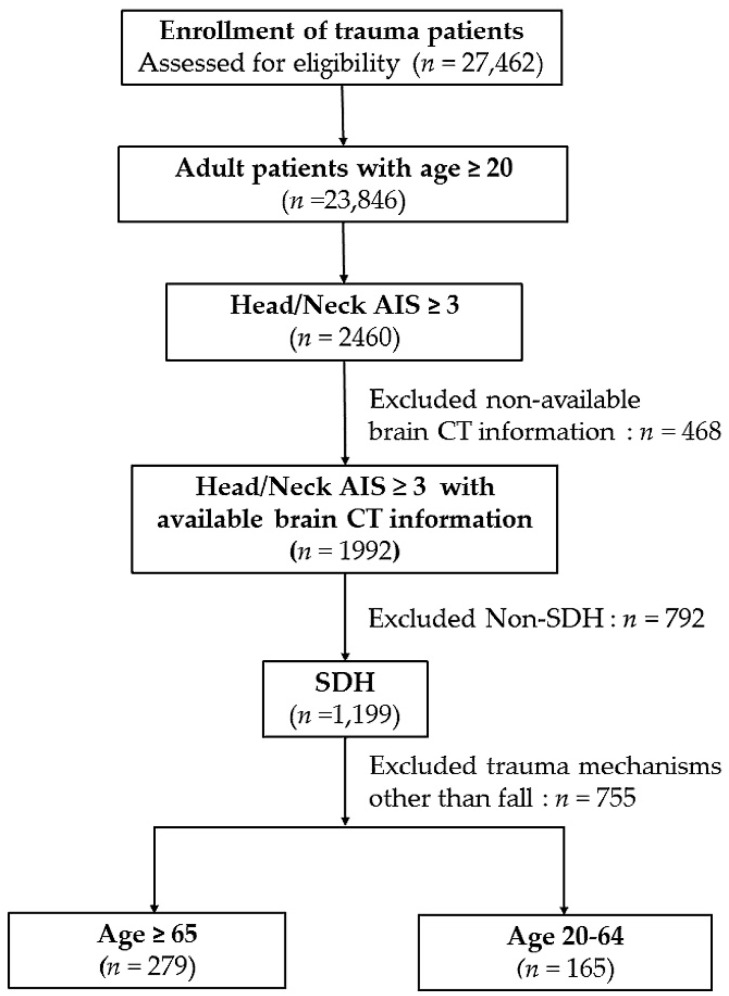
Flow chart showing the method used for selection of adult patients with acute traumatic subdural hematoma (SDH) resulting from a fall and the allocation of patients into two groups: elderly, aged ≥ 65 years, and young adults, aged 20–64 years.

A total of 444 patients with acute traumatic SDH resulting from a fall were included in this study. Patients were categorized into two groups: elderly patients (*n* = 279) and young adults (*n* = 165) (Figure 1). As shown in Table 1, there were significantly more female patients in the elderly group, compared with the young adults. The prevalence of pre-existing comorbidities including DM, HTN, CAD, CVA, and ESRD was significantly higher in elderly patients than in young adults. No significant difference was found in the rate of CHF between the two groups. The elderly patients did not have a significantly higher GCS score than the young adults, nor was there a greater percentage of elderly patients with a GCS score of ≤8, a GCS score between 9 and 12, or a GCS score ≥ 13 compared to younger patients. The rates of coagulopathy (7.2% vs. 7.9%, respectively; *p* = 0.852), ISS (median [Q1–Q3] 16 (16–20) vs. 16 (16–24), respectively; *p* = 0.434) and hematoma volume (39.0 ± 35.7 vs. 34.7 ± 30.8 mL, respectively; *p* = 0.199) were not significantly different between the elderly and the young adults.

### 3.2. Patient Outcomes

There was no difference in the rates of surgery (24.7% vs. 33.3%, respectively; *p* = 0.063) or reoperation (6.8% vs. 9.7%, respectively; *p* = 0.362) performed, LOS in the hospital (12.4 days vs. 13.7 days, respectively; *p* = 0.321) and rates of admission to the ICU (81.7% vs. 78.2%, respectively; *p* = 0.364) between the elderly and young adults. The elderly did not have a higher rate of mortality (OR 1.6, 95% CI: 0.93–2.89; *p* = 0.087) compared to young adults. However, after adjusting for gender and pre-existing comorbidities, elderly patients had a 4.0-fold higher odds ratio for mortality (95% CI, 1.06–15.45; *p* = 0.041) than young adults.

### 3.3. Risk Factors for Mortality

As shown in Table 2, the univariate logistic regression analysis demonstrated that pre-existing CAD, ESRD, hematoma volume, ISS and coagulopathy were significant risk factors for mortality in elderly patients with acute traumatic SDH resulting from a fall. In contrast, a non-isolated SDH (OR 0.9, 95% CI 0.54–1.55, *p* = 0.735) was not a significant risk factor for mortality. Pre-existing CAD (OR 3.2, 95% CI 1.09–9.69, *p* = 0.035), ESRD (OR 4.6, 95% CI 1.48–14.13, *p* = 0.008), hematoma volume (OR 1.2, 95% CI 1.11–1.36, *p* < 0.001), ISS (OR 1.3, 95% CI 1.23–1.46, *p* < 0.001) and coagulopathy (OR 4.0, 95% CI 1.47–11.05, *p* = 0.007) were all significant independent risk factors for mortality in patients with acute traumatic SDH resulting from a fall.

## 4. Discussion

In this study, we demonstrate that, after falling, the risk of mortality in elderly patients is four-fold higher than in young adults, when gender and pre-existing comorbidities are taken into accounts. Older age has long been recognized to predict further deterioration [23] and is independently associated with mortality [4] in patients with traumatic brain injuries. In this study, it is not surprising that age was related to a poor outcome in patients with acute traumatic SDH [24]. Previous studies have reported that elderly patients have worse outcomes than those under the age of 65, when undergoing conservative management [25] or surgical treatment for acute traumatic SDH [26].

Multiple factors have been reported to influence mortality in patients with traumatic acute SDH [19,27]. Patients admitted for SDH often use antiplatelet medication, implying a causal relationship between coagulopathy and this disease [28,29]. In patients with acute SDH, coagulopathy was independently associated with increased in-hospital mortality [30,31]. In a study of 248 patients with acute SDH admitted to the ICU, coagulopathy (OR 2.7, 95% CI 1.1–7.1, *p* = 0.037) was recognized as an independent predictor of in-hospital mortality [30]. In this study, we identified pre-existing CAD, ESRD, hematoma volume and coagulopathy as significant independent risk factors for mortality in patients with acute traumatic SDH. These first three variables may all be connected with the coagulopathy status of the patients.

Cardiovascular clinical trials have demonstrated that antiplatelet therapy has a major clinical benefit. Antiplatelet therapy is routinely prescribed in the secondary prevention of cardiovascular disease. Aspirin is recommended as a safe antiplatelet therapy, with the incidence of SDH being 0.02/1000 patient years in 90,689 participants [32], and there was no increase in absolute risk of SDH in patients on dual antiplatelet therapy with Aspirin and Clopidogrel [33]. However, clinical trials have reported major bleeding in 1–10% of all patients on antiplatelet therapy and this therapy is associated with significant morbidity and mortality of patients [34]. Following a trauma, the outcome of an acute SDH seems to be worse in elderly patients who used antithrombotic medication prior to injury [31]. Furthermore, the routine use of anticoagulants with heparin during hemodialysis sessions may exaggerate bleeding diathesis. It had been estimated that patients with ESRD and hemodialysis treatment are at nearly 4 times higher risk of SDH than study participants without kidney disease [35]. In the multivariate model for death, mortality was significantly associated with the presence of coagulopathy and dialysis [36]. Increased postoperative mortality was associated with dialysis (relative risk (RR) = 1.93, *p* = 0.034) and bleeding disorders (RR = 1.87, *p* = 0.003) in a study of 746 patients receiving surgical procedures for SDH [36]. In a study of 13,962 patients with a GCS of less than 15, the odds ratio for mortality following a large SDH, compared to those who had a small SDH was 3.41 (95% CI: 2.68–4.33) [15]. In contrast, the patients with isolated SDH were at a lower risk of death if they were not prescribed Warfarin or Clopidogrel [37].

As demonstrated in this study, the anatomical classifications of trauma such as ISS are well-established predictors of outcome in patients with a traumatic brain injury [2]. In a national sample of 92,030 patients who presented with SDH, an association between bony and internal organ injuries with SDH was found [4], skull fractures being the most commonly associated with SDH (19.0%), followed by spinal injuries (7.1%) and upper extremity fractures (6.8%) [4]. Mortality is increased in SDH patients with other associated injuries [4] who may have a higher ISS. In addition, the severity of brain injury can be assessed using the abbreviated injury scale (AIS), which is the fundamental score for the calculation of ISS, as the ISS score is equal to the sum of the square of three higher AIS scores in different regions of the body. If the brain was injured more severely or had accumulated more blood, the expected result would be an increased head AIS score, leading to a higher ISS. The mortality rate of participants with head AIS values of 3, 4, and 5 was 1.9%, 2.9%, and 31.1%, respectively [38]. It had been reported that the outcomes of patients with acute traumatic SDH are related to intracranial lesions [24]. The mortality rates of associated intracranial lesions were 91% for intracerebral hematoma, 87% for subarachnoid hemorrhage and 75% for contusions [25]. However, in this study, non-isolated SDH (OR 0.9, 95% CI 0.54–1.55, *p* = 0.735) was not a significant risk factor for mortality. This contradictory outcome was attributed to the fact that many severely injured patients with an isolated SDH had a large hemorrhage and therefore a high head AIS score.

Several factors have been previously reported to be associated with a poor prognosis in patients with acute SDH. These include a low baseline GCS score, pupillary abnormalities, elevated intraoperative intracranial pressure, and a large degree of midline shift revealed on CT imaging [26]. In this study, we only focus on those elderly patients with acute SDH resulting from a fall. All of the variables mentioned above may have some interaction with the variables investigated in this study, and some variables (i.e., pupillary reflex and intracranial pressure) were not registered in the trauma database. Therefore, in this study, we did not explore the contribution those variables may have made to mortality. Some bias may therefore exist in the interpretation of the results from this study. In addition, there were other limitations to this study. First, there may be a selection bias due to the retrospective design of the study. The exclusion of patients without having a brain CT image or because of unavailable CT information from the study may lead to a selection bias. Second, the patients that were declared dead upon arrival at the emergency room or at the scene of the accident were not included in the registered database, which may lead to a selection bias in the evaluation of mortality outcomes. Third, acute traumatic SDH has been traditionally considered a lesion that should be treated surgically; however, surgery for acute SDH is more controversial in elderly patients because postoperative mortality rates are reported to be high [31]. Although there is an increased use of end of life directives palliative care in the elderly than the young adult [39], in this study, we can only assume that the indications for surgery and the quality of surgery did not differ between elderly patients and young adults. Such an assumption may result in a bias in the assessment of mortality outcomes. Fourth, there is some evidence that bleeding could continue in the 24 to 48 h after head injury [40,41], and SDH has been associated with hemorrhagic progression of contusions [42]. However, the hematoma volume calculated in this study was based on the first CT images acquired at the emergency room or from the hospital that the patient was transferred from. Therefore, these results may not reflect a possible frequent dynamic change of the hematoma volume during the treatment course of this illness. Furthermore, the hematoma, ISS, and coagulopathy identified from the multivariate model may be highly related to each other variable. These interactions would lead to a limitation in the statistical analysis, since one of the presumptions in the multivariate regression is that all studied variables are independent of each other. Lastly, the real coagulopathy status of the patients was not validated in this study; thus, the association between coagulopathy and those identified independent risk factors are merely suggestive.

## 5. Conclusions

In this study, we identified that pre-existing CAD, ESRD, hematoma volume, ISS and coagulopathy were significant independent risk factors for mortality in patients with acute traumatic SDH. These results suggest that death following acute SDH is influenced by both the extent of neurological damage and the overall health of the patient at the time of injury.

## Figures and Tables

**Table 1:** Characteristics and outcomes of adult patients who sustained a subdural hemorrhage due to a fall.

Variables	Elderly (*n* = 279)	Young Adults (*n* = 165)	*p*
Age (years)	77.5 ± 7.7	49.3 ± 11.2	<0.001
Gender			<0.001
Male, *n* (%)	149(53.4)	137(83.0)	
Female, *n* (%)	130(46.6)	28(17.0)	
Comorbidities			
DM, *n* (%)	91(32.6)	29(17.6)	0.001
HTN, *n* (%)	180(64.5)	42(25.5)	<0.001
CAD, *n* (%)	31(11.1)	8(4.8)	0.024
CHF, *n* (%)	4(1.4)	2(1.2)	0.845
CVA, *n* (%)	45(16.1)	6(3.6)	<0.001
ESRD, *n* (%)	26(9.3)	5(3.0)	0.012
GCS (median, IQR)	15(11–15)	14(8–15)	0.079
1–8	54(19.4)	45(27.3)	0.053
9–12	36(12.9)	19(11.5)	0.668
13–15	189(67.7)	101(61.2)	0.162
Coagulopathy, *n* (%)	20(7.2)	13(7.9)	0.852
ISS (median, IQR)	16(16–20)	16(16–24)	0.434
Hematoma volume (mL)	39.0 ± 35.7	34.7 ± 30.8	0.199
Surgery, *n* (%)	69(24.7)	55(33.3)	0.063
Reoperation, *n* (%)	19(6.8)	16(9.7)	0.362
LOS in hospital (days)	12.4 ± 13.0	13.7 ± 13.5	0.321
ICU, *n* (%)	228(81.7)	129(78.2)	0.364
Mortality, *n* (%)	49(17.6)	19(11.5)	0.087

CAD = coronary artery disease; CHF = congestive heart failure; CI = Confidence interval; CVA = cerebral vascular accident; DM = diabetes mellitus; ESRD = end-stage renal disease; GCS = Glasgow Coma Scale; HTN = hypertension; ICU = Intensive care unit; IQR = Interquartile range; ISS = Injury Severity Score; LOS = Length of stay; OR = Odds ratio.

**Table 2:** Risk factors influencing mortality in adult patients who sustained a subdural hemorrhage in a fall, analyzed using univariate and multivariate logistic regression.

	Univariate Analysis		Multivariate Analysis	
	OR (95% CI)	*p*	OR (95% CI)	*p*
Gender (male)	0.9(0.55–1.61)	0.825	—	—
Comorbidities				
DM	0.8(0.44–1.47)	0.481	—	—
HTN	0.7(0.42–1.19)	0.189	—	—
CAD	2.4(1.13–5.09)	0.023	3.2(1.09–9.69)	0.035
CHF	—	0.999	—	—
CVA	0.6(0.22–1.49)	0.251	—	—
ESRD	2.9(1.31–6.50)	0.009	4.6(1.48–14.13)	0.008
Not an isolated SDH	1.1(0.65–1.85)	0.735	—	
Hematoma volume (mL)	1.4(1.26–1.49)	<0.001	1.2(1.11–1.36)	<0.001
ISS	1.4(1.31–1.52)	<0.001	1.3(1.23–1.46)	<0.001
Coagulopathy	5.6(2.68–11.84)	<0.001	4.0(1.47–11.05)	0.007
Surgery	1.2(0.68–2.08)	0.555	—	
Reoperation	0.9(0.34–2.45)	0.860	—	

CAD = coronary artery disease; CHF = congestive heart failure; CI = Confidence interval; CVA = cerebral vascular accident; DM = diabetes mellitus; ESRD = end-stage renal disease; HTN = hypertension; IQR = Interquartile range; OR = Odds ratio.
